# Translational Potential of the Epoxyeicosatrienoic Acid Pathway in Neurological Disorders

**DOI:** 10.3390/ijms27208990

**Published:** 2026-10-10

**Authors:** Yanwen Wang, Zuowen He

**Affiliations:** 1 Division of Cardiology, Department of Internal Medicine, Tongji Hospital, Tongji Medical College, Huazhong University of Science and Technology, Wuhan 430030, China; wangyanwentj@163.com; 2 Hubei Key Laboratory of Genetics and Molecular Mechanisms of Cardiological Disorders, Wuhan 430030, China

**Keywords:** cytochrome P450 monooxygenase, epoxyeicosatrienoic acids, soluble epoxide hydrolase, neuropsychiatric disorders

## Abstract

Neurological disorders represent a leading cause of global disability and mortality. Given that current single-target therapies often fail to halt disease progression or cross the blood–brain barrier (BBB), novel therapeutic strategies are urgently needed. Endogenous lipid networks offer promising targets. They are major structural components of the central nervous system (CNS) that provide multi-target neuroprotection essential for homeostasis and can bypass BBB restrictions naturally or via brain-penetrant small-molecule modulation. Among these lipids, arachidonic acid (AA) accounts for approximately 20% of total neuronal fatty acids. Upon cellular activation, while the cyclooxygenases (COXs) and lipoxygenases (LOXs) pathways yield pro-inflammatory mediators targeted by classic drugs, the cytochrome P450 (CYP450) cascade generates protective epoxyeicosatrienoic acids (EETs), which serve as fundamental regulators of systemic and neural homeostasis. Within the CNS, EETs act as crucial protectors of the neurovascular unit (NVU) by regulating cerebral blood flow (CBF), maintaining BBB integrity, and promoting angiogenesis. Furthermore, they actively mitigate neuroinflammation by suppressing microglial activation, reducing oxidative stress, and inhibiting neuronal apoptosis. Through these multifaceted mechanisms, alongside the enhancement of neurogenesis and synaptic plasticity, EETs effectively protect the brain against ischemic stroke, neurodegenerative diseases, and other neurological insults. Consequently, this article comprehensively reviews the physiological roles, underlying mechanisms, and regulatory factors of EETs in the nervous system. Ultimately, by highlighting the translational potential of targeted soluble epoxide hydrolase (sEH) inhibition and EET elevation, this review aims to pave the way for innovative neuroprotective therapies.

## 1. Introduction

Common neurological and neuropsychiatric disorders, including ischemic stroke, depression, autism spectrum disorder (ASD), schizophrenia, Parkinson’s disease (PD), and Alzheimer’s disease (AD), are characterized by progressive cognitive deficits, psychiatric disturbances, and severe somatic symptoms [1]. Neurological diseases remain a leading cause of health-related burden and mortality worldwide [2], often leaving patients with profound functional impairments and an inability to care for themselves. However, conventional pharmacological treatments have different mechanisms and clinical effects, but important unmet needs remain. Furthermore, the blood–brain barrier (BBB) severely restricts the central delivery of macromolecular and synthetic drugs. Consequently, there is an urgent clinical need for novel, BBB-permeable therapeutic agents capable of pleiotropically rescuing the entire neurovascular unit (NVU).

To address this challenge, endogenous lipid networks have emerged as promising targets, as they are fundamental structural components of the brain and are indispensable for the development and homeostasis of the central nervous system (CNS) [3,4,5], coupled with their ability to bypass BBB restrictions [6]. Among these lipids, arachidonic acid (AA) is particularly enriched in the brain, where it is primarily esterified within membrane phospholipids [7]. Upon cellular stimulation, AA is released from membrane phospholipids via the action of phospholipase A2 (PLA2) [8] and is subsequently metabolized through three major enzymatic cascades: cyclooxygenases (COXs), lipoxygenases (LOXs), and cytochrome P450 (CYP) pathways [9]. While the COXs and LOXs pathways predominantly generate pro-inflammatory eicosanoids that can exacerbate tissue injury, the CYP epoxygenase pathway (primarily the CYP2C and CYP2J subfamilies) catalyzes the epoxidation of AA to generate four epoxyeicosatrienoic acid (EET) regioisomers: 5,6-EET, 8,9-EET, 11,12-EET, and 14,15-EET. However, these EETs are swiftly hydrolyzed by soluble epoxide hydrolase (sEH) into their corresponding, biologically less active diols, dihydroxyeicosatrienoic acids (DHETs) [10,11,12].

The AA–CYP450–EET–sEH metabolic cascade is a fundamental regulator of systemic homeostasis [13]. Through the generation of bioactive EETs, this pathway exerts potent anti-inflammatory, anti-apoptotic, and vasodilatory effects across various tissues [9,14,15]. Within the CNS, these CYP-derived lipid mediators act as vital paracrine and autocrine modulators of the NVU, coordinating reciprocal interactions between vascular, glial, and neuronal compartments [16]. Functionally, EETs bridge the gap between vascular restoration and neuronal survival by facilitating microvascular remodeling and vasodilation to optimize cerebral perfusion, while simultaneously preserving BBB integrity [16,17,18]. Concurrently, they modulate glial cell activation, tilting the microenvironment toward a neuroprotective phenotype that actively suppresses neuroinflammatory propagation [19,20]. Furthermore, these mediators support synaptic maintenance and network plasticity, shielding neural circuitry from progressive excitotoxic and oxidative damage [20,21,22]. This multifaceted neurovascular protection underscores the therapeutic potential of targeting the AA–CYP450–EET–sEH axis to mitigate severe neurological insults and maintain the functional homeostasis of neural networks.

This article comprehensively reviews the current knowledge regarding the roles and underlying mechanisms of the CYP/EET/sEH axis in treating major neurological disorders. Additionally, we will critically discuss the translational pros and cons of genetic knockout versus pharmacological interventions, the divergent responses to treatment timing across different disease stages, sex-dependent variations in drug efficacy, and the emerging impact of cross-organ metabolic crosstalk on brain homeostasis. Ultimately, by highlighting the evidence and limitations of targeted sEH inhibition and EET elevation, this review aims to identify testable directions for neuroprotective research.

## 2. Metabolism and Subcellular Compartmentalization of the AA Epoxygenase Pathway in the Nervous System

**Figure 1:**
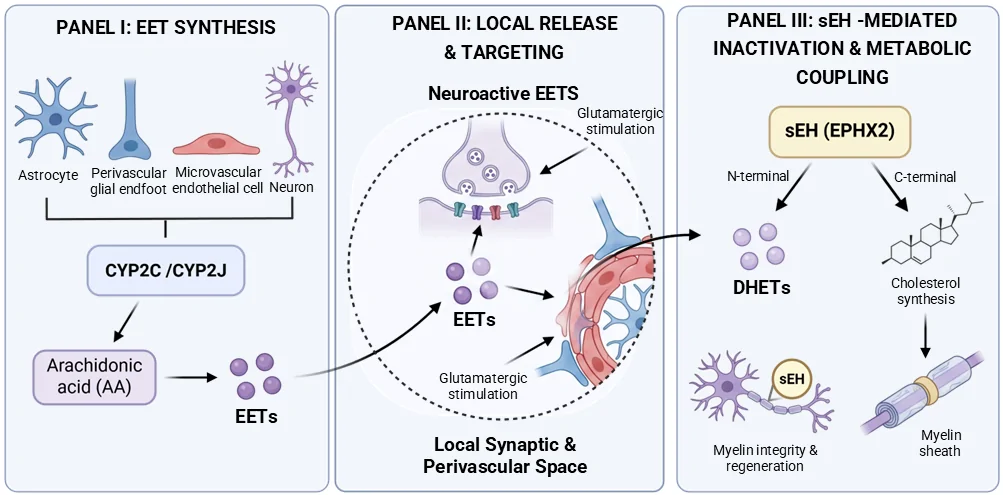
CYP-derived EET pathway in the nervous system. Panel I (EET Synthesis): In the neurovascular unit, AA is released from membrane phospholipids and converted by CYP2C/CYP2J epoxygenases in astrocytes, perivascular glial endfeet, microvascular endothelial cells, and neurons to generate EETs. Panel II (Local Release and Targeting): Glutamatergic stimulation triggers the local release of neuroactive EETs into the synaptic and perivascular spaces, where they act on nearby cellular targets. Panel III (Inactivation and Metabolic Coupling): The C-terminal hydrolase activity of sEH converts EETs to DHETs; the N-terminal domain has lipid phosphatase activity. AA: arachidonic acid; CYP: cytochrome P450; EETs: epoxyeicosatrienoic acids; sEH: soluble epoxide hydrolase; DHETs: dihydroxyeicosatrienoic acids. Created in BioRender. He, Z. (2026) https://BioRender.com/kgy9f2t.

Endogenous lipid networks are essential for CNS homeostasis and represent a viable therapeutic target. Within this lipid pool, AA, a 20-carbon ω-6 polyunsaturated fatty acid, is a major component of cell membranes. It is particularly abundant in the CNS, where it constitutes approximately 20% of the neuronal fatty acid pool and remains primarily esterified within membrane phospholipids [7]. Under physiological and pathophysiological conditions, such as neuronal activation, ischemia, or neuroinflammation, intracellular calcium (Ca^2+^) levels rise, activating cytosolic phospholipase A2 (cPLA2). This enzyme cleaves AA from the sn-2 position of membrane phospholipids, releasing free AA into the intracellular space [8]. Upon liberation from membrane phospholipids, free AA is subject to metabolism via three predominant enzymatic cascades: the COX, LOX, and CYP pathways [9]. While the COXs and LOXs pathways typically generate pro-inflammatory eicosanoids like prostaglandins and leukotrienes, the CYP epoxygenase pathway is primarily responsible for the biosynthesis of EETs [9,16] (Figure 1).

The synthesis of these lipid mediators exhibits cell-type specificity within both the central and peripheral nervous systems [16,23]. Key epoxygenase isoforms, including rodent CYP2C11 and rodent CYP2J isoforms, CYP2C11 is expressed in astrocytes, perivascular glial endfeet, and microvascular endothelial cells [24,25,26], and rodent CYP2J isoforms are expressed in peripheral ganglia neurons [23]. Glutamatergic synaptic transmission promotes the rapid release of neuroactive EETs into the gliovascular and synaptic spaces, a process mediated by metabotropic glutamate receptor activation and subsequent intracellular Ca^2+^ mobilization in astrocytes [27].

The biological half-life and spatial diffusion of EETs are primarily constrained by their rapid hydration to vicinal DHETs [13]. This reaction is catalyzed by sEH, a homodimeric, bifunctional enzyme encoded by the EPHX2 gene, which possesses C-terminal epoxide hydrolase activity and N-terminal lipid phosphatase activity [28,29,30]. Although sEH is ubiquitously distributed in cerebral tissues, with pronounced expression in astrocytes, oligodendrocytes, and neuronal somata [19,31], immunocytochemical analyses have revealed predominant axonal localization of this enzyme in primary cortical, sympathetic, and sensory neurons [32]. This precise subcellular distribution supports a model in which sEH functions as a local metabolic gate at the growth cone, thereby tightly regulating the microenvironmental gradients of bioactive lipid epoxides [32]. Furthermore, the N-terminal phosphatase domain of sEH has been implicated in cerebral cholesterol biosynthesis and homeostasis [30]. While neuronal cholesterol synthesis is critical for remyelination in chronic lesions, astrocyte-derived cholesterol also supports remyelination. Given this essential role of local cholesterol synthesis in myelin integrity and axonal regeneration, the dual catalytic functions of sEH—the N-terminal phosphatase involved in cholesterol precursor metabolism and the C-terminal hydrolase regulating EET signaling—may serve to couple cellular lipid homeostasis with neuroregenerative programs [28].

## 3. Neuroprotective Roles of EETs in the Nervous System

**Figure 2:**
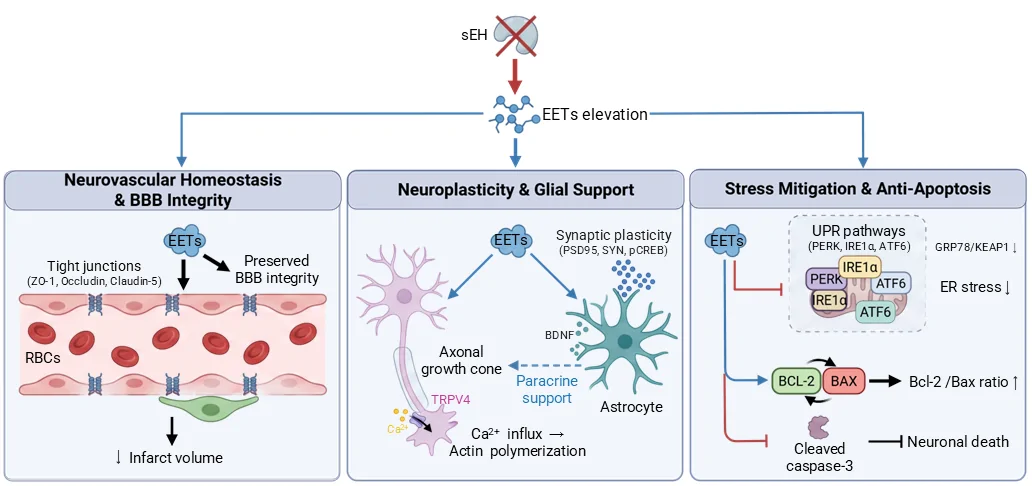
Schematic overview of the neuroprotective actions of EETs and the effects of sEH inhibition in the nervous system. This figure illustrates three interconnected protective axes. Left (Neurovascular Homeostasis and BBB Integrity): EETs preserve BBB integrity by maintaining tight junction proteins (ZO-1, occludin, claudin-5), reducing infarct volume and limiting RBC extravasation. Center (Neuroplasticity and Glial Support): 14,15-EET promotes axonal growth cone remodeling via TRPV4-mediated Ca^2+^ influx and actin polymerization, enhances synaptic plasticity markers (PSD95, SYN, pCREB); elevated EETs stimulate astrocytic BDNF release to provide paracrine neuronal support in vivo. Right (Stress Mitigation and Anti-Apoptosis): Elevated EETs suppress ER stress sensors (PERK, IRE1α, ATF6) and downregulate GRP78/KEAP1, while increasing the Bcl-2/Bax ratio and reducing cleaved caspase-3, thereby blocking apoptotic neuronal death. Together, these pathways mediate the pleiotropic neuroprotective effects of EET-related signaling, which are amplified by pharmacological or genetic sEH inhibition. Notably, while this schematic focuses on EET-related signaling, sEH acts on multiple epoxy fatty acids, so its blockade simultaneously elevates other protective neuroactive lipids beyond EETs. sEH: soluble epoxide hydrolase; EETs: epoxyeicosatrienoic acids; BBB: blood–brain barrier; RBCs: red blood cells; TRPV4: transient receptor potential vanilloid 4; Ca^2+^: calcium; BDNF: brain-derived neurotrophic factor; UPR: unfolded protein response; ER: endoplasmic reticulum. Created in BioRender. He, Z. (2026) https://BioRender.com/3ytw0w0.

EETs function as pleiotropic lipid mediators that coordinate neuroprotective programs, including the preservation of neurovascular coupling, the promotion of synaptic plasticity, and the inhibition of neuronal apoptosis. This section systematically discusses the multifaceted actions of EETs under physiological and pathophysiological conditions, with particular emphasis on their roles in maintaining neurovascular homeostasis, regulating glial–neuronal networks, and counteracting cellular stress. The therapeutic potential of augmenting EETs via sEH inhibition is discussed throughout (Figure 2).

### 3.1. Maintenance of Neurovascular Homeostasis

NVU, an integrated structural ensemble linking cerebral hemodynamics with BBB function, is essential for CNS homeostasis. Within this microenvironment, endogenous EETs function as vasoactive and endothelial-protective mediators. Under physiological conditions, they promote cerebral vasodilation, stimulate angiogenesis, and modulate platelet adhesion [33]. The protective efficacy of the CYP450–EET axis is evident in genetic models. Endothelial-targeted overexpression of CYP2J2 in transgenic mice elevates 14,15-EET biosynthesis. Following global cerebral ischemia induced by bilateral common carotid artery occlusion (BCCAO), this increase preserves regional cerebral blood flow (CBF) and microvessel density, suppresses reactive oxygen species (ROS) production, limits infarct expansion, and attenuates apoptosis via activation of the pro-survival PI3K/AKT signaling cascade. Consistent with these in vivo findings, exogenous EET administration or CYP2J2 transfection directly rescues astrocytes from oxygen-glucose deprivation (OGD)-induced apoptosis through parallel pathways [17], underscoring the pleiotropic nature of EETs in neurovascular preservation.

Preventing EET degradation through genetic or pharmacological targeting of sEH has emerged as a promising interventional strategy. Notably, the vascular effects of sEH blockade exhibit a nuanced profile. While genetic sEH deletion reduces infarct size and increases local CBF post-ischemia [34], pharmacological inhibition with AUDA-BE or t-AUCB restricts ischemic damage and optimizes the local EET–DHET ratio without significantly altering global or regional CBF, an observation that extends to streptozotocin (STZ) induced diabetic models [35,36,37]. This dissociation suggests that whereas permanent sEH gene ablation may promote macrovascular reperfusion and structural remodeling, the acute neuroprotection conferred by pharmacological sEH inhibitors operates through non-hemodynamic mechanisms.

Among these non-hemodynamic actions is the direct stabilization of the BBB. Composed primarily of specialized brain endothelial cells, the BBB regulates molecular transit and restricts peripheral immune infiltration [38]. During cerebral ischemia, degradation of tight junction proteins triggers BBB disruption and cerebral edema. Post-ischemic administration of the selective sEH inhibitor TPPU counteracts this structural failure. By preserving the expression of critical tight junction proteins, ZO-1, occludin, and claudin-5, TPPU maintains BBB integrity, thereby resolving edema and limiting total infarct area [39]. In the rat DAI hyperglycemia model, sEH inhibition protects BBB integrity by upregulating tight junction protein expression and downregulating inflammatory cytokine levels both in vivo and in vitro [40]. sEH inhibitors and sEH gene knockout mitigated BBB leakage in both mouse [41] and rat [42] models of AD. This barrier-stabilizing effect extends to systemic inflammatory insults. In models of sepsis-associated encephalopathy (SAE) induced by cecal ligation and puncture (CLP), aberrant sEH upregulation in the brain correlates with perivascular microglial activation, BBB leakage, and anxiety-like behaviors. TPPU treatment reverses this endothelial dysfunction and mitigates the associated neurobehavioral deficits [43].

From a translational perspective, the capacity of the EET–sEH axis to preserve BBB integrity independently of hemodynamic changes holds clinical relevance. A major limitation in acute ischemic stroke management is the narrow therapeutic window for reperfusion therapies (e.g., thrombolysis), which carry a high risk of hemorrhagic transformation driven by ischemia-induced BBB breakdown. Because brain-penetrant sEH inhibitors can fortify endothelial tight junctions and rescue ischemic tissue without inducing abrupt hemodynamic alterations, they represent a promising adjunctive strategy. Whether sEH inhibition can safely extend a thrombolytic time window or improve human outcomes has not been established. This approach could theoretically extend the thrombolytic time window, mitigate reperfusion injury, and provide a versatile pharmacological tool for managing complex neurovascular and systemic inflammatory disorders.

### 3.2. Promotion of Neuroplasticity and Glial–Neuronal Crosstalk

Neural network remodeling, encompassing axonal outgrowth, synaptogenesis, and glial–neuronal communication, is fundamental to CNS development and repair. Endogenous EETs, particularly those generated via the CYP450 epoxygenase pathway, act as drivers of this structural and functional plasticity. Experimental evidence indicates a regioisomer-specific effect: 14,15-EET uniquely stimulates axonal elongation in central and peripheral neurons and nerve growth factor (NGF)-induced neurite outgrowth, whereas 8,9-EET and 11,12-EET lack comparable neuroregenerative activity [32,44]. At the mechanistic level, 14,15-EET promotes neurite outgrowth through a TRPV4-dependent mechanism. The TRPV4 inhibitor HC067047 blocks the effects of 14,15-EET on PC12 cell neurite outgrowth. Furthermore, 14,15-EET increases cytosolic calcium ion concentration, and this increase is inhibited by HC067047, suggesting that 14,15-EET contributes to neurite outgrowth through calcium influx via TRPV4 [44]. Accordingly, TRPV4 blockade abolishes 14,15-EET-mediated structural outgrowth, while inhibition of baseline CYP450 activity with ketoconazole impairs intrinsic neurite extension. [44]. Beyond these direct neuronal actions, EETs coordinate intercellular crosstalk. During ischemic challenges such as oxygen-glucose deprivation and reperfusion (OGD/R), 14,15-EET stimulates astrocytes to synthesize and secrete brain-derived neurotrophic factor (BDNF), establishing a paracrine survival network that protects adjacent neurons [45]. Furthermore, direct intracerebral infusion of 14,15-EET reduces amyloid-beta (Aβ) deposition by enhancing lysosomal biogenesis in astrocytes [46], indicating a regulatory role within the neuro–glial microenvironment.

Given that the neurotrophic potential of endogenous EETs is constrained by their rapid hydrolysis, sEH inhibition offers a strategy to amplify neuroplasticity. sEH is widely distributed in neuronal somas, dendrites, and axons [35], and is abundantly expressed in astrocytes [47,48]. Pharmacological sEH blockade with selective inhibitors (e.g., 4-PCO or TUPS) potentiates 14,15-EET-driven axonal outgrowth in cultured sensory and cortical neurons [32], and the sEH inhibitor AUDA enhances excitatory glutamate receptor transmission in the prefrontal cortex in mouse brain slices [49]. In the 5XFAD mouse model, chronic low-dose co-treatment with TPPU and rivastigmine—but not monotherapy—enhanced protein levels of synaptic plasticity markers, including the pCREB ratio, BDNF, and PSD95. In contrast, no substantial improvements in synaptic plasticity markers were observed in the senescence-accelerated mouse prone 8 (SAMP8) model, where the same co-treatment strategy instead reduced neuroinflammatory gene expression [50]. In experiments using 5XFAD mice, sEH inhibitor UB-SCG-74 treatment significantly improved cognition and synaptic plasticity, outperforming donepezil, a standard AD drug, and ibuprofen, an anti-inflammatory drug [51].

Notably, while pharmacological sEH inhibition consistently limits pathological astrogliosis, the effects of global sEH genetic knockout can be context-dependent, occasionally exacerbating acute immune responses in specific lipopolysaccharide (LPS)-stimulated models [52], showing that genetic and pharmacological interventions should not be treated as interchangeable. The EET–sEH axis is also linked to BDNF/TrkB signaling across various neurological pathologies. In murine models of depression, astrocyte-specific sEH knockout promotes ATP release [53], while preventive TPPU administration or systemic sEH deletion averts stress-induced depressive-like behaviors by restoring BDNF levels and TrkB phosphorylation in the hippocampus and prefrontal cortex [54]. Similarly, following middle cerebral artery occlusion (MCAO), sEH inhibition engages the BDNF/TrkB pathway in the peri-infarct region, driving sensorimotor recovery, an effect abolished by the TrkB antagonist ANA12 [55]. Furthermore, in a valproic acid-induced animal model of ASD, sEH gene knockout ameliorates social deficits and fear memory extinction deficits. The underlying mechanism is associated with restoring dysregulated AMPK–mTOR signaling in the prefrontal cortex and repairing synaptic plasticity [56].

The ability of brain-penetrant sEH inhibitors to simultaneously promote neuronal rewiring and modulate glial reactivity presents an attractive therapeutic paradigm. By metabolically amplifying endogenous EETs, these small molecules stimulate astrocytic BDNF release and enhance synaptic resilience. This approach circumvents the pharmacokinetic limitations and poor BBB penetrance associated with direct exogenous neurotrophin administration, and these preclinical findings motivate tests of whether appropriately exposed sEH inhibitors improve neuroplasticity or depressive symptoms in humans.

### 3.3. Mitigation of Cellular Stress and Cell Death Pathways

At the subcellular level, the accumulation of misfolded proteins and oxidative insults in neuropsychiatric and neurodegenerative disorders triggers endoplasmic reticulum (ER) stress. Prolonged proteotoxic stress initiates terminal apoptotic cascades, resulting in irreversible neuronal loss. Endogenous EETs function as lipid mediators that buffer these stress responses and maintain proteostasis. However, their rapid hydrolysis by sEH limits their intrinsic protective capacity. Accordingly, therapeutic amplification of EET signaling via genetic or pharmacological sEH blockade represents a strategy to intercept cell death at multiple upstream and downstream checkpoints.

Elevated EET levels attenuate the ER stress response [57,58,59]. In murine models of PD, both genetic sEH knockout and treatment with TPPU suppress hyperactivation of the three canonical ER stress sensor pathways—PERK, IRE1α, and ATF6—within the striatum of 1-methyl-4-phenyl-1,2,3,6-tetrahydropyridine (MPTP)-intoxicated mice [57]. Critically, this intervention halts activation of the ER-specific caspase-12 in the substantia nigra, severing the molecular link between unresolved ER stress and apoptosis initiation [58]. This upstream resilience is also observed in AD pathology. In Aβ-infused models, TPPU administration downregulates the ER stress chaperone GRP78(BiP) and KEAP1 in the hippocampus. This effect is mechanistically coupled to activation of the Nrf2/HO-1 antioxidant axis, indicating that sEH blockade both restores protein folding capacity and counteracts oxidative damage [59].

When cellular damage progresses toward terminal execution, enhanced EET bioavailability exerts anti-apoptotic effects across diverse disease etiologies. In cellular models of PD, aberrant sEH upregulation occurs in PARK2-mutant induced pluripotent stem cell (iPSC)-derived neurons; subsequent sEH blockade with TPPU reduces expression of cleaved caspase-3, a final apoptotic effector [57]. Similarly, the sEH inhibitor APAU curtails rotenone-induced apoptotic signaling, specifically the JNK/c-Jun axis and active caspase-3, in N27 dopaminergic cells [60], while in vivo TPPU treatment attenuates pro-caspase-9 levels in the substantia nigra of MPTP-treated mice [58]. This modulation of the intrinsic apoptotic machinery extends to cerebrovascular and AD models. Following permanent MCAO in rats, post-injury TPPU administration mitigates structural brain damage by downregulating the pro-apoptotic effectors BAX and caspase-3 while upregulating the anti-apoptotic guardian BCL-2 [39]. Similar Bcl-2/Bax rebalancing, alongside attenuation of p53 and caspase-9/-3 cleavage, is observed in the hippocampi of Aβ-challenged AD mice following TPPU treatment [59]. Notably, while the sEH inhibitor AUDA modulates the transcriptome of apoptosis-related genes in stroke-prone spontaneously hypertensive rats, corresponding changes at the protein level were not consistently detected [61], suggesting that lipid-mediated interventions may exert pre-translational regulatory control before widespread structural damage manifests.

Progressive neuronal loss driven by sustained ER stress and apoptosis constitutes the pathological core of PD and AD. Because brain-penetrant sEH inhibitors concurrently quench upstream unfolded protein response (UPR) pathways and dismantle downstream caspase-driven execution, these experimental findings provide a rationale for further clinical testing.

## 4. Translational Potential of the EET–sEH Axis in Neurological and Psychiatric Disorders

**Figure 3:**
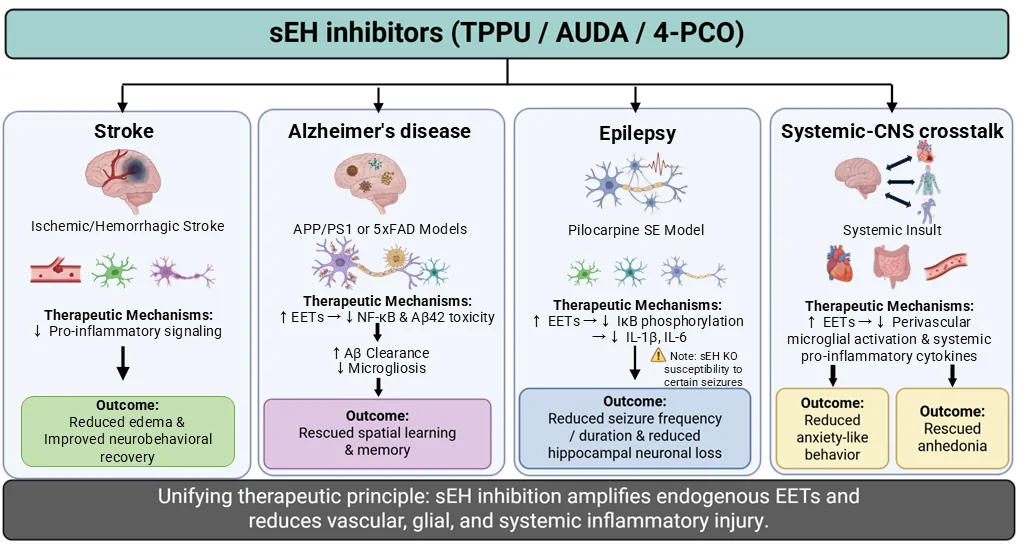
Therapeutic applications of sEH inhibition across neurological and psychiatric disorders. This figure summarizes the preclinical findings of brain-penetrant sEH inhibitors (TPPU, AUDA, 4-PCO) in four major disease categories. Stroke: In ischemic and hemorrhagic stroke models, sEH inhibition reduces pro-inflammatory signaling, decreases infarct/edema, and improves neurobehavioral recovery. AD: In APP/PS1 or 5xFAD models, sEH blockade increases EETs, suppresses NF-κB and Aβ42 toxicity, enhances Aβ clearance, reduces microgliosis, and rescues spatial learning and memory. Epilepsy: In pilocarpine-induced status epilepticus models, sEH inhibition elevates EETs, inhibits IκB phosphorylation, lowers IL-1β and IL-6, and reduces seizure frequency/duration and hippocampal neuronal loss (note that global sEH knockout may increase seizure susceptibility). Systemic–CNS crosstalk: In systemic insult models (cardiac arrest, SAE, chronic pain-induced anhedonia), sEH inhibition raises EETs, suppresses perivascular microglial activation and systemic pro-inflammatory cytokines, and alleviates anxiety-like behaviors. The unifying therapeutic principle is that sEH inhibition amplifies endogenous EETs alongside other bioactive epoxy fatty acids, thereby mitigating vascular, glial, and systemic inflammatory injury. sEH: soluble epoxide hydrolase; EETs: epoxyeicosatrienoic acids; AD: Alzheimer’s disease; Aβ: Amyloid-β; IκB: inhibitor of nuclear factor kappa B; IL-1β: interleukin-1 beta; IL-6: interleukin-6; SAE: sepsis-associated encephalopathy. Created in BioRender. He, Z. (2026) https://BioRender.com/tphkhpr.

The neuroprotective mechanisms detailed above position the EET–sEH metabolic axis as a pharmacologically tractable target. Brain-penetrant sEH inhibitors offer a means to augment endogenous EETs, thereby circumventing the pharmacokinetic limitations inherent to direct exogenous lipid administration. This section evaluates the predominantly preclinical intervention evidence for targeting this lipid network across acute cerebrovascular pathologies, chronic neurodegenerative conditions, epileptic hyperexcitability, and systemically driven mood disorders (Figure 3).

### 4.1. Interventional Strategies in Cerebrovascular Pathologies

Cerebrovascular diseases, including both ischemic and hemorrhagic strokes, share a pathological trajectory driven by acute vascular compromise and secondary neuroinflammation. Human genetic evidence supports the clinical relevance of the EET–sEH axis in this context: polymorphisms within or near the EPHX2 gene (encoding sEH) are associated with increased susceptibility to ischemic stroke [62]. This linkage underscores the potential value of targeting this pathway to preserve cerebrovascular integrity.

Pharmacological sEH blockade confers structural and molecular protection in acute ischemic stroke models. Following AUDA treatment, Wistar Kyoto rats subjected to MCAO displayed mild cerebral infarction and olfactory bulb (OB) degeneration alongside better olfactory performance, with the OB showing reduced pro-inflammatory mediators and enhanced antioxidant processes [63]. The therapeutic efficacy of sEH inhibition extends to hemorrhagic stroke models. In a mouse model of collagenase-induced intracerebral hemorrhage (ICH), both genetic sEH knockout and prophylactic treatment with the inhibitor AUDA [64] or TPPU [65] blunt microglial-mediated neuroinflammation and rescue peri-hematomal neurons. Similarly, in a mouse model of experimentally induced subarachnoid hemorrhage (SAH), sEH ablation alleviates vascular inflammation and cerebral edema, correlating with improved neurobehavioral recovery [66].

The cellular loci mediating these protective effects reflect the dual nature of the neurovascular network. Although sEH expression in the CNS is prominently observed in reactive astrocytes, its abundant expression in systemic endothelial cells suggests that the cerebrovascular endothelium may represent an equally critical site of EET-mediated protection. Thus, modulating sEH activity at this endothelial–glial interface may constitute a dual-action strategy, simultaneously reinforcing endothelial integrity and attenuating glial immune responses across ischemic and hemorrhagic stroke subtypes.

Current acute stroke management is constrained by the narrow therapeutic window of thrombolytics for ischemic events and the absence of neuroprotective therapies for hemorrhagic strokes. sEH inhibitors have shown endothelial and glial effects in animal models. Their effect on bleeding risk and clinical outcomes across cerebrovascular indications requires direct testing.

### 4.2. Experimental Evidence in AD

AD is characterized by Aβ deposition, tau hyperphosphorylation, and neuroinflammation [67]. Human tissue observations suggest sEH is upregulated in brains [68]. This observation suggests that restoring the EET–sEH axis may confer disease-modifying effects. At the cellular level, EET elevation via the sEH inhibitor TPPU attenuates nuclear factor kappa B (NF-κB) signaling and counteracts Aβ42-induced neurotoxicity in human neuroblastoma cell lines [69]. In amyloid precursor protein/presenilin 1 (APP/PS1) transgenic mice, genetic sEH ablation promotes an anti-inflammatory astrocytic phenotype, facilitating Aβ clearance and rescuing behavioral deficits [70]. Similarly, using sEH inhibitors in 5xFAD and SAMP8 mice reduced Aβ plaque accumulation and tau hyperphosphorylation pathology, suppressed neuroinflammation, and restored spatial learning and memory [68]. In Aβ-induced AD mice, TPPU alleviated spatial learning and memory deficits, suppressed neuroinflammation, and inhibited neuronal apoptosis via stabilizing EETs [59]. Notably, the protective influence of the EET–sEH axis may extend to early developmental stages. Maternal TPPU administration during gestation and lactation improves cognitive outcomes in 5xFAD offspring at two months of age, reducing microgliosis and tau hyperphosphorylation independently of changes in Aβ42 levels [71]. This finding suggests a role for lipid mediators in conferring early neural resilience beyond amyloid burden.

The therapeutic versatility of sEH inhibition is also evident in mixed-etiology neurodegeneration. In STZ-induced diabetes-associated AD-like pathology, sEH inhibitor TPPU suppresses metabolic neuroinflammation, an effect synergistically amplified by co-administration of DHA [72]. Recent drug development has yielded dual-target inhibitors that simultaneously block sEH and AChE. Long-term, low-dose administration of these compounds—or co-administration of separate sEH and AChE inhibitors—has shown efficacy in suppressing neuroinflammation while enhancing synaptic plasticity and memory retention, as demonstrated in the SAMP8 mouse model (for the sEH/AChE dual inhibitor) and in both the SAMP8 and 5xFAD mouse models (for combined sEH and AChE inhibition) [21,50].

Current AD therapies offer symptomatic relief or face safety concerns regarding neurovascular inflammation. Brain-penetrant sEH inhibitors—particularly when formulated as dual-target agents—address the root neurodegenerative drivers by promoting Aβ clearance and resolving glial inflammation, thereby representing a promising disease-modifying strategy.

### 4.3. Mitigating Neural Network Instability in Epilepsy

Status epilepticus (SE) triggers neuroinflammation, a driver of neural network hyperexcitability. Following pilocarpine-induced SE, sEH expression is upregulated from day seven post-induction [73]. Pharmacological blockade with sEH inhibitors (AUDA, TPPU) preserves endogenous EET levels. At the molecular level, this stabilization suppresses SE-induced neuroinflammation by inhibiting inhibitor of nuclear factor kappa B (IκB) phosphorylation and downregulating pro-inflammatory cytokine transcription (IL-1β, IL-6). AUDA treatment reduces both seizure frequency and duration in SE mice [73]. In the KA-induced chronic epileptic model, TPPU exhibited neuroprotective properties by reducing neuronal apoptosis and neuroinflammation, and by suppressing Akt/mTOR pathway activation [74]. These effects are reproducible across models: in rat lithium chloride/pilocarpine-induced SE, TPPU blunts glial activation and inflammatory mediator release in the hippocampus and frontal cortex, mitigating neuronal loss and seizure frequency [75].

In contrast to pharmacological inhibition, global sEH knockout mice exhibit exacerbated susceptibility to epileptogenesis compared to wild-type controls [73]. The divergent findings in experimental models [73,75] may reflect developmental compensation, cell and tissue context, intervention timing, or different effects on the hydrolase and phosphatase domains. By resolving the cycle of neuroinflammation and hyperexcitability, targeted pharmacological sEH inhibitors warrant further preclinical and clinical study as possible adjuncts in epilepsy.

### 4.4. Modulating Systemic–Brain Crosstalk in Depression and Comorbidities

Beyond primary neurological pathologies, the EET–sEH axis modulates the brain’s response to systemic insults, a phenomenon that reflects systemic-to-brain crosstalk. In a mouse model of cardiac arrest/cardiopulmonary resuscitation (CA/CPR), global cerebral ischemia following cardiac arrest precipitates hippocampal neuronal death and memory loss. Post-arrest, cerebral expression of the neurotoxic cytokines TNF-α and IL-1β is elevated. The sEH inhibitor 4-PCO reveals a nuanced immunomodulatory mechanism: rather than simply suppressing microglial activation, it upregulates production of the anti-inflammatory cytokine interleukin-10 (IL-10) [76]. This profile indicates that sEH inhibition reprograms reactive microglia toward a neuroprotective phenotype, curbing secondary neuronal death.

This immunomodulation extends to systemic infections. SAE is characterized by sustained microglial activation driving cognitive and neuropsychiatric deficits. In SAE models, TPPU alleviates anxiety-like behaviors by suppressing perivascular microglial activation [43]. Similarly, in LPS-induced neuroinflammation, TPPU attenuates sensory hyperalgesia by elevating endogenous EETs and disrupting inflammasome assembly [77]. The multi-organ distribution of the EET–sEH axis links somatic distress to psychiatric outcomes. In murine models of chronic pain-induced anhedonia (a core depressive symptom), sEH levels are aberrantly elevated across a systemic network—including the medial prefrontal cortex (mPFC), hippocampus, spinal cord, liver, kidneys, and intestinal tissues—accompanied by a surge in circulating pro-inflammatory factors. Systemic TPPU administration neutralizes these peripheral cytokines and abolishes anhedonic and depressive-like behaviors [19].

Experimental findings across cardiac arrest, sepsis, and chronic pain models suggest that sEH modulation may influence systemic and central inflammatory responses. Whether these mechanisms translate into effective treatment of human mood symptoms or secondary brain injury has not been established.

## 5. Systemic Regulation and Crosstalk Between EETs in Other Organs and the Neurological System

**Figure 4:**
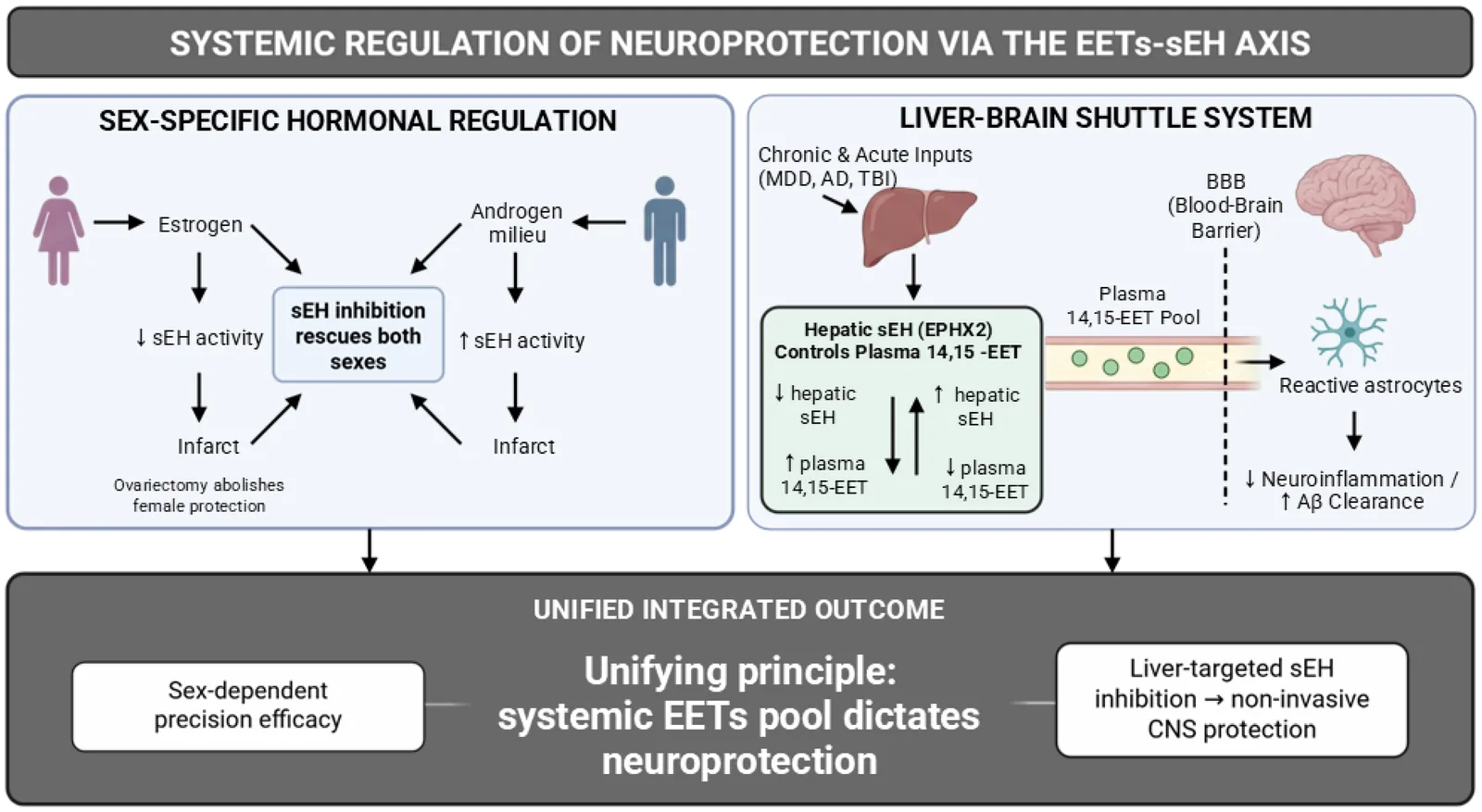
Systemic regulation of neuroprotection via the EET–sEH axis. This figure illustrates two principal peripheral regulatory mechanisms that modulate central nervous system outcomes investigated mainly in cell and animal models. Left (Sex-specific Hormonal Regulation): Estrogen suppresses sEH activity and reduces infarct size in females, whereas an androgen milieu increases sEH activity and infarct size. Ovariectomy abolishes female protection, while pharmacological sEH inhibition rescues both sexes, indicating that sex hormones modulate the basal activity and therapeutic responsiveness of the EET–sEH axis. Right (Liver–Brain Shuttle System): Hepatic sEH controls the circulating plasma 14,15-EET pool. Upregulation of hepatic sEH depletes plasma 14,15-EET, whereas downregulation elevates it. Plasma 14,15-EET crosses the BBB to promote reactive astrocytes, reduce neuroinflammation, and enhance Aβ clearance. This shuttle operates in both chronic conditions. The systemic EET pool dictates neuroprotection through sex-dependent precision efficacy; liver-targeted sEH inhibition offers a non-invasive strategy for CNS protection. sEH: soluble epoxide hydrolase; EETs: epoxyeicosatrienoic acids; BBB: blood–brain barrier; Aβ: Amyloid-β; MDD: major depressive disorder; AD: Alzheimer’s disease; TBI: traumatic brain injury; CNS: central nervous system. Created in BioRender. He, Z. (2026) https://BioRender.com/sas154x.

Research on the neuroprotective functions of the EET–sEH axis has historically adopted a CNS-centric perspective. Emerging evidence, however, indicates that neural lipid homeostasis is subject to regulation by systemic factors. This section addresses the modulation of the central EET–sEH metabolic network by two such variables: sex-dependent hormonal dimorphism and inter-organ lipid crosstalk. Understanding these systemic influences may inform non-invasive pharmacokinetic strategies that leverage peripheral organs to support central neurological functions (Figure 4).

### 5.1. Sex-Dependent Dimorphism in the EET–sEH Axis

Biological sex and circulating sex hormones influence the basal activity and therapeutic responsiveness of the EET–sEH axis. Within the brain, sEH expression is regulated by sex hormones and is repressed by estrogen [78]. Accordingly, sexual dimorphism is observed under pathological stress. In vitro, primary brain endothelial cells from male mice exhibit higher baseline sEH activity, lower endogenous EET levels, and greater cellular damage following OGD compared with female-derived cells. Pharmacological sEH inhibition rescues endothelial viability in both sexes, effectively neutralizing this biological disparity [79]. This dimorphism translates to in vivo cerebrovascular events. Following MCAO, female mice exhibit suppressed sEH activity, superior CBF, and reduced infarct volumes relative to males. These female-specific neuroprotective advantages are abolished by ovariectomy or in global sEH knockout models, confirming the role of estrogen–sEH signaling in neuroprotection [78].

Metabolic and neurodegenerative progression also exhibit sex-specific divergence. In response to a 12-week high-sucrose diet, male mice show upregulation of neuroinflammatory and neurodegenerative genes in the hippocampal vasculature, whereas female mice display a downregulatory protective response. Targeted sEH inhibition reverses this aberrant transcriptomic profile selectively in males [80]. Lipidomic profiling further indicates that male brain lipidomes undergo more extensive remodeling following chronic sEH inhibitor intervention than those of females [81]. Dysregulation of sEH in AD patients exhibits sex- and disease-stage-dependent differences [82]. Systemic lipid interventions can also induce sex-dependent epigenetic reprogramming. When wild-type females mated with heterozygous 5xFAD males are treated with TPPU during pregnancy and lactation, offspring of both sexes show reduced tau phosphorylation, diminished microgliosis, and rescued memory decline at two months of age. However, this maternal intervention upregulates specific AD risk factors (e.g., GFAP, EGR1, chitinase-like 1 (*Chil1*) and sirtuin 2 (*Sirt2*)) selectively in female progeny [71]. Collectively, these findings indicate that preclinical evaluations and clinical translations of EET-targeted therapies should account for sex-dependent metabolic differences.

### 5.2. The Liver–Brain Lipid Axis

Conventional approaches to neuropsychiatric treatment have focused on engineering brain-penetrant molecules. However, peripheral lipid metabolism exerts a substantial influence on CNS health. Human associative findings and experimental animal studies suggest that hepatic lipid metabolism may influence circulating EET-related measures and brain outcomes.

In chronic psychiatric and neurodegenerative conditions, this hepatic–cerebral pathway plays a critical role. Increased sEH protein expression was observed in both the cortex and liver tissues of patients with major depressive disorder (MDD), bipolar disorder (BD), and SZ [83]. In murine depression animal models, targeted hepatic sEH knockout produces antidepressant effects, whereas liver-specific sEH overexpression exacerbates depressive-like behaviors. By modulating circulating plasma 14,15-EET levels, the liver determines the availability of this lipid messenger to interact with mPFC astrocytes to resolve central neuroinflammation [84]. This endocrine-like transport is also relevant in chronic neurodegeneration. In aging and 5xFAD models of AD, targeted hepatic sEH knockdown elevates plasma 14,15-EET, which subsequently crosses the BBB to modulate central Aβ metabolism and cognitive function—a neuroprotective outcome replicated by direct intracerebroventricular (ICV) infusion of 14,15-EET [85].

Beyond chronic regulation, the liver–brain axis also functions as an acute rescue mechanism following neurological trauma. In murine traumatic brain injury (TBI) models, hepatic sEH (EPHX2) activity undergoes adaptive downregulation immediately after trauma. This transient suppression is inversely correlated with a systemic surge in circulating plasma 14,15-EET concentrations [6]. Pharmacokinetic tracing confirmed that, in adult C57BL/6J mice, 14,15-EET-d11 was detectable in plasma and cortex within 1 min following intracarotid administration. Upon CNS entry under traumatic TBI conditions, they promote polarization of reactive astrocytes toward an astrocytic state associated with protection. Overexpression of hepatic sEH abolishes the compensatory elevation of peripheral 14,15-EET, thereby altering the reported astrocytic response and exacerbating post-traumatic neurological deficits [6].

The discovery of this liver-to-brain lipid shuttle suggests a pharmacological strategy in which drugs that do not themselves cross the BBB may still confer CNS protection by selectively inhibiting peripheral (hepatic) sEH. These animal studies raise the hypothesis that by elevating circulating plasma EETs—which act as endogenous penetrant vectors—peripheral sEH inhibitors can bypass central pharmacokinetic barriers, establishing a non-invasive paradigm for treating both acute and chronic central neurological disorders.

## 6. Discussion

This review has synthesized the pleiotropic neuroprotective functions of the EET–sEH metabolic axis across a range of neurological and psychiatric disorders. As endogenous lipid mediators, EETs contribute to BBB integrity, cerebral hemodynamic regulation, and synaptic plasticity. At the cellular level, sustained EET bioavailability—achieved primarily through modulation of astrocytic sEH activity—reshapes the neuro–glial microenvironment. This intervention concurrently attenuates neuroinflammation, alleviates ER stress, interrupts apoptotic signaling, and promotes BDNF-dependent survival pathways. Furthermore, it is crucial to distinguish direct EET-mediated effects from the broader consequences of sEH inhibition. Because sEH metabolizes multiple epoxy fatty acids—including those derived from docosahexaenoic acid (DHA) and eicosapentaenoic acid (EPA)—the in vivo neuroprotection conferred by pharmacological sEH inhibitors likely reflects the synergistic preservation of a broader spectrum of epoxy fatty acids (EpFAs), rather than selective EET elevation alone. Future studies utilizing selective EpFA infusions or precise lipidomic profiling are required to dissect these overlapping mechanisms.

Translating these mechanistic insights into clinical application requires optimization of the spatiotemporal profile of sEH inhibition. Preclinical studies have examined several treatment windows for sEH inhibitors, with efficacy reported across prophylactic regimens (administered days to weeks before MCAO or intracerebral hemorrhage) [37,61,64], acute peri-interventional dosing (during ischemia or before reperfusion) [36,86], and delayed post-injury administration (up to 24 h after stroke onset) [35]. Early-life interventions also suggest disease-modifying potential: maternal TPPU administration during gestation and lactation rescues cognitive deficits in 5xFAD offspring [71]. To maximize therapeutic benefit, recent drug discovery efforts have increasingly embraced polypharmacology. A dual sEH/AChE inhibitor [21] and, separately, combined low-dose administration of distinct sEH and AChE inhibitors [50] have produced favorable memory-related outcomes in AD mouse models. Neither approach has established clinical efficacy. Nevertheless, direct comparisons of dosing schedules remain limited owing to variability in experimental protocols; future studies should systematically define optimal dose regimens and therapeutic time windows for specific disease contexts.

Genetic EPHX2 deletion and pharmacological sEH inhibition are distinct interventions. Constitutive global deletion removes the protein throughout development and across tissues, eliminating both the C-terminal epoxide hydrolase and N-terminal phosphatase functions. Over the past few decades, the vast majority of developed sEH inhibitors have indeed targeted the C-terminal hydrolase activity, and for many of these inhibitors, selectivity data against the N-terminal phosphatase have not been routinely assessed [87]. Global sEH knockout has been shown to attenuate disease progression in specific models of PD, depression, hemorrhagic and ischemic stroke [34,54,57,64,66], and to promote beneficial astrocytic polarization in APP/PS1 mice [70]. In other contexts, however, it has yielded detrimental outcomes, including exacerbating neuroinflammation after LPS challenge [52], inducing baseline anxiety-like behaviors [88], and increasing susceptibility to epileptogenesis [73]. These discordant findings suggest that lifelong, whole-body gene deletion may reflect developmental compensation, tissue-specific actions, or distinct enzymatic functions, as well as experimental variables, such as model choice, dose, and timing.

## 7. Future Directions

Human translation remains at an early stage. EC5026 has published phase 1a single-dose and food-effect safety/pharmacokinetic data in healthy volunteers [89]; a phase 1/phase 2 study in Parkinson’s disease (STEP, NCT07142044) is designed to evaluate safety, pharmacokinetics, target engagement, and exploratory efficacy, as clinical benefit has not yet been established. GSK2256294 has phase 1 safety/pharmacodynamic data [90] and was tested in a small phase 1b randomized aneurysmal subarachnoid hemorrhage trial (10 treated, 9 placebo) [91]. No study-drug-related adverse events were reported in that trial, and serum—but not cerebrospinal fluid—EET/DHET ratios increased; neurological efficacy was not established [91]. These findings support further trials, not current clinical use for neurological or psychiatric disease.

## 8. Limitations

Several challenges must be addressed to facilitate clinical translation. A major hurdle in CNS drug development is the reliance on invasive cerebrospinal fluid sampling to confirm central target engagement, a practice limited by ethical and practical constraints. Key limitations include heterogeneous animal models and treatment schedules, limited evidence separating EET-specific effects from other epoxy fatty acids, and uncertain extrapolation from lifelong EPHX2 deletion to hydrolase-selective drugs. In addition, future trial designs should incorporate patient stratification based on key systemic variables—particularly sex-dependent hormonal dimorphism, given the inhibitory effect of estrogen on sEH expression.

## 9. Literature Search Strategy

A comprehensive literature search was conducted through the PubMed database for studies published up to September 2026 to identify relevant research. The search strategy employed combinations of the following keywords: sEH OR EET AND relevant section topics. Inclusion criteria were limited to peer-reviewed articles published in English. In addition, the reference lists of the included articles were manually screened to further confirm that the literature content matched the scope of this review.

## Data Availability

No new data were created or analyzed in this study. Data sharing is not applicable to this article.
